# The evolution of ageing in cooperative breeders

**DOI:** 10.1002/evl3.307

**Published:** 2022-12-01

**Authors:** Jan J. Kreider, Boris H. Kramer, Jan Komdeur, Ido Pen

**Affiliations:** ^1^ Theoretical Research in Evolutionary Life Sciences, Groningen Institute for Evolutionary Life Sciences University of Groningen Groningen 9747 AG The Netherlands; ^2^ Behavioural and Physiological Ecology, Groningen Institute for Evolutionary Life Sciences University of Groningen Groningen 9747 AG The Netherlands

**Keywords:** Ageing, cooperative breeding, inclusive fitness, individual‐based simulations, kin selection, life history, lifespan, reproductive queueing, senescence, sociality

## Abstract

Cooperatively breeding animals live longer than their solitary counterparts. This has been suggested for birds, mole rats, and social insects. A common explanation for these long lifespans is that cooperative breeding evolves more readily in long‐lived species because lower mortality reduces the rate of territory turnover and thus leads to a limitation of breeding territories. Here, we reverse this argument and show that—rather than being a cause for its evolution—long lifespans are an evolutionary consequence of cooperative breeding. In evolutionary individual‐based simulations, we show that natural selection favors a delayed onset of senescence in cooperative breeders, relative to solitary breeders, because cooperative breeders have a delayed age of first reproduction as helpers wait in a reproductive queue to obtain breeder status. Especially long lifespans evolve in cooperative breeders in which queue positions depend on the helpers’ age rank among the helpers within the breeding territory. Furthermore, we show that lower genetic relatedness among group members leads to the evolution of longer lifespans. This is because selection against higher mortality is weaker when mortality reduces competition for breeding between relatives. Our results link the evolutionary theory of ageing with kin selection theory, demonstrating that the evolution of ageing in cooperative breeders is driven by the timing of reproduction and kin structure within breeding territories.

**Impact Summary**

Across animals, there appears to be an association between sociality and longevity. For instance, social birds, mole rats, and insects are more long‐lived than solitary species, and also humans have longer lifespans than solitary primates. However, we lack evolutionary explanations for these patterns as currently the evolutionary theory of ageing has mainly been applied to solitary organisms. We here present an evolutionary computational model to explain lifespan variation between solitary and social and within social organisms. Furthermore, by investigating effects of the presence of relatives on the evolution of ageing, we incorporate kin selection arguments into the evolutionary theory of ageing. The evolution of sociality and ageing have long been recognized as evolutionary paradoxes, and thus as central challenges for evolutionary biology.

The evolution of sociality is associated with changes in life history, especially lifespan (Kramer et al. 2021; Kreider et al. 2021; Pen and Flatt 2021). In birds (Arnold and Owens 1998; Downing et al. 2015), mole rats (Healy 2015; Williams and Shattuck 2015) (though not in mammals in general; Lukas and Clutton‐Brock 2012a; Thorley 2020), and probably in non‐eusocial social insects (Séguret et al. 2016), cooperatively breeding species often have longer lifespans than solitary species. Cooperative breeders are group‐living organisms in which some group members temporarily do not reproduce and take on a helper role, whereas other individuals are breeders (Rubenstein and Abbot 2017; Taborsky et al. 2021). This contrasts with eusocial organisms in which helpers belong to a worker or soldier caste distinct from breeders and are unable to reproduce (Crespi and Yanega 1995; Boomsma 2013; Boomsma and Gawne 2018). Just as cooperative breeders, also the reproductive castes of eusocial organisms are extraordinarily long‐lived; however, queens and kings (in termites) typically do not only outlive solitary organisms but also their workers by several orders of magnitude (Keller and Genoud 1997; Kramer and Schaible 2013). Thus, both cooperative breeding and eusociality are typically associated with the occurrence of long lifespans.

The prevalence of long lifespans in cooperative breeders has been interpreted as evidence in favor of the so‐called “life history hypothesis” of cooperative breeding (Hatchwell and Komdeur 2000; Pen and Weissing 2000; Kokko and Lundberg 2001; Kokko and Ekman 2002). This hypothesis posits that particular life‐history traits, such as low adult mortality, facilitate the evolution of cooperative breeding because lower mortality reduces the rate of territory turnover. If access to breeding territories is restricted, it can be beneficial for individuals to remain near their natal territory and help raise offspring of relatives (“indirect fitness benefits”) (Hamilton 1964a, 1964b; Hatchwell 2009; Cornwallis et al. 2010; Lukas and Clutton‐Brock 2012b) and/or to queue for a breeding territory with a chance to inherit a breeding position (“direct fitness benefits”), even if this requires alloparental care towards nonrelatives (Leadbeater et al. 2011; Zöttl et al. 2013; Quiñones et al. 2016; Kingma 2017). Thus, the logic of the “life history hypothesis” implies that cooperative breeders are long‐lived because the longevity of their solitary ancestors played a causal role in the evolution of cooperative breeding.

However, the logic may also be reversed—rather than being a cause of cooperative breeding, long lifespans could be a consequence of it—although both of these directions of causality are not mutually exclusive. This seems to follow from Hamilton's classical theory on the evolution of senescence (Hamilton 1966), which demonstrates that the strength of natural selection against higher mortality is maximal and constant until the age of first reproduction and declines with age afterward. Consequently, a delayed age of first reproduction implies the evolution of a delayed onset of senescence and thus longer lifespans. In cooperative breeders, sexually mature helpers typically have to wait for an extended period in a reproductive queue and therefore have a delayed age of first reproduction (Downing et al. 2015). As a result, cooperatively breeding species should evolve longer lifespans than otherwise similar solitary species. However, Hamilton's model did not explicitly consider effects of sociality (Kramer et al. 2016), hence the need for a formal model of the evolution of ageing in cooperative breeders.

Here, we present an evolutionary individual‐based simulation model to derive predictions for the evolution of ageing in cooperatively breeding organisms. The model represents a population of individuals whose lifespans evolve due to the accumulation of mutations with age‐specific effects on survival, as in Medawar's mutation accumulation theory of ageing (Medawar 1952). We simulated the evolution of ageing both in solitarily and cooperatively breeding organisms, representing a broad range of biological systems (Fig. 1). As the productivity benefits of helpers, that is, the increase in the reproductive output of the dominant breeder caused by the presence of helpers, can vary between cooperatively breeding species, we investigated how such productivity benefits affect the evolution of ageing. In cooperative breeders, reproductive queues may consist of highly related individuals but also of nonrelated individuals. Therefore, we evaluated the effect of kin structure within breeding territories on the evolution of ageing in cooperative breeders.

**Figure 1:**
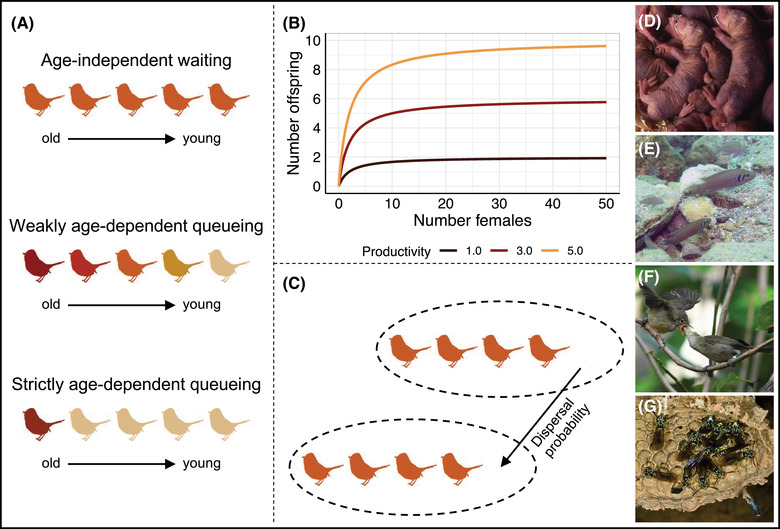
Overview of model scenarios and examples of cooperatively breeding animals. (A) Three different scenarios of waiting/queueing for obtaining breeder status. Red color intensity is proportional to the probability of individuals to become the breeder relative to other competing individuals. Age of individuals decreases from left to right. Under age‐independent waiting, all individuals have the same probability to obtain breeder status, independently of their age. Under weakly age‐dependent queueing, older individuals are more likely to become the new breeder. Under strictly age‐dependent queueing, the oldest individual always becomes the new breeder. (B) Different scenarios of “maximum productivity” as a function of number of females in a breeding territory. The higher the productivity, the more offspring are produced, leading to larger groups. (C) Depending on the dispersal probability, helpers can disperse from their natal breeding territory at independence and join a queue in another breeding territory (breeding territories = dashed ovals). (D–G) Examples of cooperative breeders to which the model predictions might apply. (D) The naked mole rat (*Heterocephalus glaber*) is a long‐lived rodent that lives in closely related family groups and exhibits age‐dependent reproductive queueing (Clarke and Faulkes 1997; Sherman and Jarvis 2002; O'Riain and Faulkes 2008; Van der Westhuizen et al. 2013) (copyright: Chris Faulkes). (E) The Lake Tanganyika princess (*Neolamprologus pulcher*) is a cooperatively breeding cichlid with low relatedness within groups and size‐dependent reproductive queueing (Dierkes et al. 2005; Stiver et al. 2005; Wong and Balshine 2011) (copyright: Dario Josi). (F) The Seychelles warbler (*Acrocephalus sechellensis*) has low within‐group relatedness and age‐independent waiting (Richardson et al. 2002; Eikenaar et al. 2007; Groenewoud et al. 2018) (copyright: Charli Davies). (G) The tropical hover wasp (*Liostenogaster flavolineata*) has high relatedness within groups and age‐dependent reproductive queueing (Shreeves and Field 2002; Sumner et al. 2002; Bridge and Field 2007; Cronin and Field 2007) (copyright: David Baracchi).

## Methods

### SOLITARY AND COOPERATIVE BREEDING SCENARIOS

We developed an evolutionary individual‐based simulation model. The model represents a population with a fixed number of breeding territories *N* (for all model parameters and their default values, see Table 1). Each breeding territory is initialized with one breeding female. We modeled seven different breeding systems. (1) *Solitary breeding*: Females disperse at independence and compete for empty breeding territories. Females that fail to obtain a breeding territory die during that breeding season. (2) *Solitary breeding with age‐independent waiting*: Females disperse at independence to become floaters that wait for breeding territories to become empty. The probability of females to occupy an empty breeding territory is independent of their age. (3) *Solitary breeding with weakly age‐dependent queueing*: Females again disperse at independence to become floaters. However, now older females are more likely to obtain a breeding territory than younger females. (4) *Solitary breeding with strictly age‐dependent queueing*: Again, females disperse to become floaters but now the oldest female always has priority to obtain an empty breeding territory. (5) *Cooperative breeding with age‐independent waiting*: Females become helpers who wait locally in a breeding territory with a chance to inherit the breeder position after the breeder's death. A new breeder is randomly selected from all helpers in the breeding territory. (6) *Cooperative breeding with weakly age‐dependent queueing*: Females queue locally in a breeding territory. Their probability to inherit the breeding position increases with their relative age in the breeding territory. (7) *Cooperative breeding with strictly age‐dependent queueing*: Females again queue locally in a breeding territory. Upon a breeder's death, the oldest helper always becomes the new breeder.

**Table 1:** Model parameters. If parameter ranges are given, the exact parameter value is stated in the figure caption. For further parameter exploration, see the Supporting Information

Parameter	Value	Meaning
*N*	1000	Maximum population size
*t*_end_	80,000	Number of time steps
*d*	0.0–1.0	Dispersal rate
*c*	20	Maximum age
*m*	0.02	Mutation rate
*μ*	−0.009	Mutation bias
*σ*	0.018	Mutational effect size
*g*	0.8	Initial gene value
*q*	0.0, 0.8, 1.0	Age‐dependency of waiting/queueing
*a*	1.0–5.0	“Maximum productivity”
*b*	0.5	Parameter for diminishing return function
*F*_max_	50,000	Maximum queue length

In all three cooperative breeding scenarios, helpers can additionally be selected randomly to become a breeder if a breeding territory other than their own becomes empty. Furthermore, in all cooperative breeding scenarios, helpers disperse at independence with probability *d* to become a helper in another breeding territory than their natal breeding territory. To reduce model complexity, we assume that only females can become helpers, as in many arthropod cooperative breeders (Davies et al. 2016). Males always disperse from their natal breeding territory and mate randomly with females from the entire population.

### GENETICS

Individuals have diplodiploid genetics and carry two homologous genes for each age from age 0 to the maximum age *c*. Thus, individuals have 2*c* genes. Each gene is associated with a gene value that ranges from 0.0 to 1.0. The gene values of the homologous genes are averaged to determine an age‐specific survival probability. Gene values are mutated with a genome‐wide mutation rate *m* at the time of emergence of a new individual. We assume that mutational effects are biased towards negative values, and thus lower survival to model accumulation of age‐specific deleterious mutations (Medawar 1952). If a mutation occurs, then each of the maternally or paternally inherited *c* gene values is mutated by separately sampling a normal distribution with a mean of *μ* < 0 (“mutation bias”) and a standard deviation of *σ* (“mutational effect size”). If a mutation causes the gene values to fall below their lower limit of 0.0 or exceed their upper limit of 1.0, they are set back to the respective limit. The maternally and paternally inherited gene values can be recombined during gene transmission to offspring. We varied the default parameters for mutation rates, mutation biases, and mutational effect sizes in the Supporting Information (Fig. S1). All gene values were initialized with values of *g* (see Table 1).

### SURVIVAL

Females survive or die depending on their genetically determined age‐specific survival probability. Additionally, females die when they reach the maximum age *c*. We varied the default maximum age in the Supporting Information (Figs. S2, S3). To not confound the evolution of lifespans of females with selection on male survival (males do not help), we assume that males do not express genes for survival and that they always die after one time step.

### REPRODUCTIVE WAITING/QUEUEING

In model scenarios 2–7, floaters or helpers wait/queue globally or locally in a breeding territory to obtain breeder status. Floaters and helpers (within breeding territories) are sorted according to their age (from old to young), and a weight for each floater or helper determines how likely a given individual becomes a breeder. The weight of the *i*th floater or helper is $w_{i}=q^{i-1}$, where *q* is a parameter that determines how strongly the probability of the individual to become a breeder depends on its age rank among the other floaters or helpers within the breeding territory. If *q* = 0, then the oldest individual always obtains the breeding territory (“strictly age‐dependent reproductive queueing”). If 0 < *q* < 1, then relatively older individuals are more likely to obtain the breeder position (“weakly age‐dependent reproductive queueing”). If *q* = 1, then the probability to obtain the breeder position is independent of age (“age‐independent waiting”).

### REPRODUCTION

We assume that the number of offspring produced by a breeder increases with the number of helpers in the breeding territory. The expected number of offspring is modeled with the diminishing return function

(1) $$R=\frac{aF}{1+bF},$$

where *F* is the number of females in the breeding territory, *a* is a “maximum productivity” parameter that determines the maximum number of offspring at large group sizes, and *b* is a further parameter that determines the rate at which the maximum is approached. The number of offspring *R* is stochastically rounded to an integer. Offspring are produced with even sex ratios. Females mate at independence with one random male. We limited the number of females in a queue (in global queues of floaters as well as in local queues within breeding territories) to *F*_max_ for computational reasons. This maximum queue length was never reached in any of the simulations.

### MODEL ANALYSIS

We implemented the model in C++ and compiled it with g++ 4.8.5. We analyzed and visualized model results in R 4.1.0 (R Core Team 2021) using the packages *ini* (Dias 2018), *gridExtra* (Auguie 2017), *scales* (Wickham and Seidel 2020), *tidyverse* (Wickham et al. 2019), *cowplot* (Wilke 2019), *ggpubr* (Kassambara 2020), and *MetBrewer* (Mills 2021). We ran simulations until time point *t*_end_. At this point, the simulations had reached an evolutionary quasi‐equilibrium at which mean lifespans no longer changed systematically over time (Fig. S4). We calculated evolved lifespans as the sum of the cumulative products of age‐specific survival probabilities (“life expectancy”). To obtain estimates for relatedness between breeders and helpers, we gave individuals two selectively neutral homologous genes that were randomly mutated without mutation bias. We then used the mean of the two homologous genes from all breeders and one random helper per breeding territory to calculate relatedness within breeding territories as the covariance between the breeder and helper gene values divided by the product of the standard deviations of the breeder and helper gene values.

## Results

### THE EVOLUTION OF AGEING IN SOLITARY AND COOPERATIVE BREEDERS

**Figure 2:**
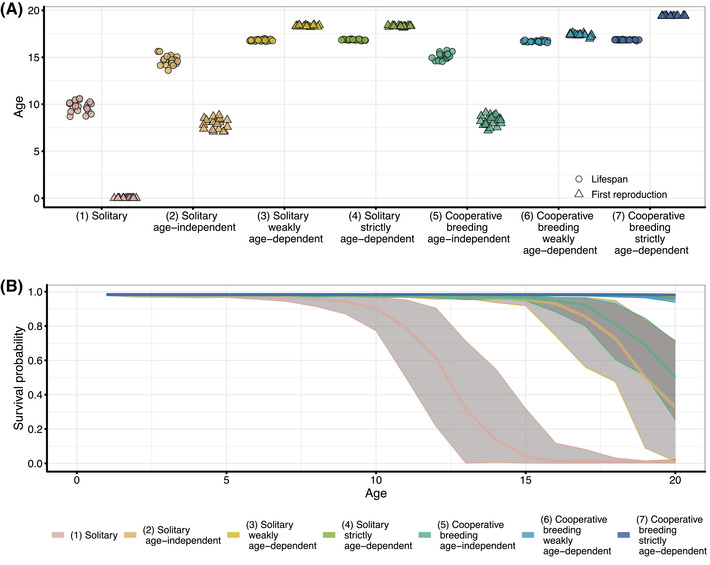
The evolution of ageing in solitary and cooperative breeders. (A) Evolved lifespans (circles) and age of first reproduction (triangles) for different solitary and cooperative breeding scenarios. In the age‐dependent reproductive queueing scenarios, average age of first reproduction exceeds evolved lifespans. However, as Figure S5 shows, survivorship until the age of first reproduction is high (more than 70% of the individuals in the population). Data points (*n* = 20) are the population mean at the end of a replicate simulation. (B) Age‐specific survival probabilities for different solitary and cooperative breeding scenarios. Bold lines represent the mean of age‐specific survival probabilities and gray‐shaded areas the range across replicate simulations. Lines for the age‐dependent queueing scenarios overlap. Parameters: *d* = 1.0 (dispersal rate), *a* = 2.5 (“maximum productivity”)

If nonbreeding individuals can inherit a breeding territory later in their life, either through waiting/queueing for an unoccupied breeding territory globally as floaters in the solitary breeding scenarios or locally within a breeding territory as helpers in the cooperatively breeding scenarios, longer lifespans evolve than in the absence of queueing (“(1) Solitary”). Longer lifespans coincide with an increase in the age of first reproduction (Fig. 2). In cooperative breeders, queueing positions within breeding territories may depend on the helpers’ age relative to that of other helpers in the group (see examples in Fig. 1). Under such age‐dependent queueing (“(6) Cooperative breeding weakly age‐dependent” and “(7) Cooperative breeding strictly age‐dependent”), longer lifespans evolve and the age of first reproduction increases relative to age‐independent waiting (“(5) Cooperative breeding age‐independent”). This is also the case under age‐dependent queueing in solitary breeding systems (“(3) Solitary weakly age‐dependent” and “(4) Solitary strictly age‐dependent”) compared to solitary breeding systems with no such age effects (“(2) Solitary age‐independent”). Furthermore, evolved lifespans are numerically similar between solitary and cooperative breeders with the same age dependency of waiting/queueing, despite some statistical differences between them (two‐sample *t*‐test: (2) Solitary age‐independent vs. (5) Cooperative breeding age‐independent: means 14.68 vs. 15.10, *P* < 0.01; (3) Solitary weakly age‐dependent vs. (6) Cooperative breeding weakly age‐dependent: means 16.82 vs. 16.70, *P* < 0.001; (4) Solitary strictly age‐dependent vs. (7) Cooperative breeding strictly age‐dependent: means 16.86 vs. 16.86, *P* = 0.62). The increase of lifespans through age‐dependent reproductive queueing compared to age‐independent waiting is even stronger when maximum lifespans are increased from 20 to 40 (Fig. S2).

### THE EFFECT OF PRODUCTIVITY ON GROUP SIZE AND THE EVOLUTION OF AGEING

**Figure 3:**
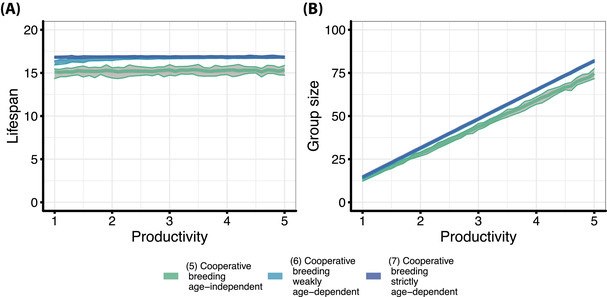
The effect of “maximum productivity” on group size and the evolution of ageing in cooperative breeders. (A) Evolved lifespans depending on “maximum productivity” in different cooperative breeding scenarios. “Maximum productivity” is a model parameter that determines the number of offspring produced at large group sizes. (B) The effect of “maximum productivity” on group size. Lines for the two age‐dependent queueing scenarios overlap. We ran each “maximum productivity” between *a* = 1.0 and *a* = 5.0 with steps of 0.1 (*n* = 20). Bold lines represent the mean evolved lifespan and gray areas the range of evolved lifespans across replicate simulations. Parameters: *d* = 0.5 (dispersal rate)

In cooperatively breeding species, helpers typically have a positive effect on the breeder's reproduction or on offspring survival (Hatchwell 2004; Doerr and Doerr 2007; Canestrari et al. 2011; Brouwer et al. 2012; Preston et al. 2016; Koenig et al. 2019). However, it has been shown that these positive helper effects diminish at large group sizes (Schwarz 1988; Kramer et al. 2014). Therefore, we modeled reproductive output of breeders as an increasing function with diminishing returns with respect to the number of helpers present, converging toward a “maximum productivity,” the maximum number of offspring that can be produced at large group sizes. Increased “maximum productivity” leads to larger groups. Additionally, groups are larger under age‐dependent reproductive queueing than under age‐independent reproductive waiting due to increased lifespans. However, in all scenarios of cooperative breeding, irrespective of whether helpers are in age‐dependent reproductive queues or whether their probability to breed is independent of age, “maximum productivity” has only a small effect on the evolution of lifespans (Fig. 3). This is also the case under no and maximum dispersal (Fig. S6).

### THE EFFECT OF DISPERSAL ON RELATEDNESS WITHIN BREEDING TERRITORIES AND THE EVOLUTION OF AGEING

**Figure 4:**
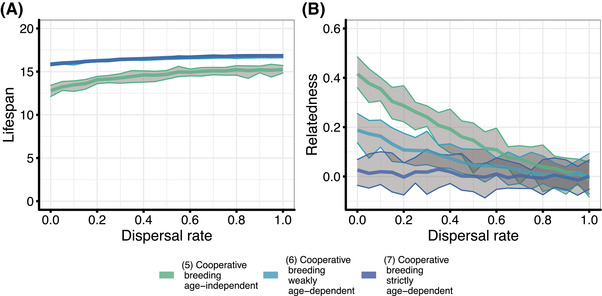
The effect of dispersal on relatedness within breeding territories and the evolution of ageing in cooperative breeders. (A) The effect of dispersal on evolved lifespans in different cooperative breeding scenarios. Lines for the two age‐dependent queueing scenarios overlap. (B) The effect of dispersal rate on relatedness between breeders and helpers in different cooperative breeding scenarios. We varied dispersal rates between *d* = 0 and *d* = 1 in steps of 0.05 (*n* = 20). Bold lines represent the mean and gray areas the range across replicate simulations. Parameters: *a* = 2.5 (“maximum productivity”)

In cooperatively breeding species, relatedness between individuals that share a breeding territory can vary (see examples in Fig. 1). We consequently allowed offspring either to be philopatric and become a helper in the natal breeding territory or to disperse and become a helper in another breeding territory. We estimated relatedness between the breeder and a random helper from the same breeding territory. Under age‐independent waiting, higher dispersal rates result in lower relatedness within breeding territories and lead to the evolution of longer lifespans than under lower dispersal rates (Fig. 4). In both cases of age‐dependent reproductive queueing, in contrast, relatedness within breeding territories is relatively low. This is because under age‐dependent reproductive queueing, age of first reproduction increases compared to age‐independent waiting, and this increases the number of generations of females waiting in the reproductive queue, thus diluting relatedness. Lifespans are consequently not as strongly affected by dispersal rate.

## Discussion

The “life history hypothesis” of cooperative breeding explains the prevalence of long lifespans in cooperative breeders by proposing that long lifespans facilitate the evolution of cooperative breeding (Hatchwell and Komdeur 2000; Pen and Weissing 2000; Kokko and Lundberg 2001; Kokko and Ekman 2002). Using evolutionary individual‐based simulations, we show that, conversely, long lifespans in cooperative breeders can also evolve as a consequence of cooperative breeding rather than being a cause for its evolution. Our results do not contradict the proposition that long lifespans facilitate the evolution of cooperative breeding—a claim that has also received formal support (Pen and Weissing 2000). Indeed, it seems likely that longevity and sociality are mutually reinforcing (Alexander et al. 1991; Carey 2001; Carey and Judge 2001; Korb and Heinze 2021). However, our model also shows that evolved lifespans hardly differ between solitary organisms and cooperatively breeding organisms in fully outbred populations, under the same age dependency of waiting/queueing. In both cases, queueing individuals compete exclusively with unrelated individuals. This shows that not cooperative breeding per se, but instead the structuring of queues and the timing of first reproduction are the main determinant of the evolved onset of senescence in both solitary and cooperatively breeding organisms. In queueing systems, age of first reproduction, in turn, is influenced by lifespan as longer lifespans decrease the rate of territory turnover and thus delay the onset of reproduction. Consequently, there is positive feedback between age of first reproduction and evolved lifespans in queueing systems.

The argument that long lifespans evolve as a consequence of cooperative breeding is not unprecedented, although different mechanisms have been proposed to cause these long life spans. For instance, recent work suggests that breeders live longer because they can afford to reduce their costly parental investment in the presence of helpers (Hammers et al. 2019, 2021; Downing et al. 2021). In our model, in contrast, longer lifespans evolve in cooperative breeders due to the delayed age of first reproduction (Hamilton 1966). Both of these mechanisms are not mutually exclusive, and the simultaneous presence of both might increase lifespans even more than each mechanism on its own.

Comparative studies have yielded somewhat ambiguous findings on the association between lifespan and cooperative breeding—some found longer lifespans in cooperative breeders compared to solitary species (Arnold and Owens 1998; Downing et al. 2015; Healy 2015; Williams and Shattuck 2015) and some did not (Lukas and Clutton‐Brock 2012a; Thorley 2020). Our results show large variation of evolved lifespans within solitarily breeding and cooperatively breeding organisms, depending on the mechanisms of waiting/queueing. It might consequently be informative to consider the waiting/queueing mechanisms as predictors in future interspecific lifespan comparisons.

Our model furthermore shows that positive effects of helping on reproductive output only have a minor effect at best on the evolution of lifespans in cooperatively breeding organisms, although maximum productivity clearly affected group size. In eusocial species, colony size positively correlates with the divergence of queen and worker lifespans (Kramer and Schaible 2013). However, in cooperative breeders there seems to be only a small effect of group size on the evolution of ageing, as our model suggests. This is probably because the productivity benefits of helping to raise related offspring are to some extent counteracted by the simultaneous increase of competition for breeder positions (West et al. 2002).

In contrast to the lack of productivity effects, the presence of relatives within breeding territories does affect the evolution of lifespans. Higher within‐territory relatedness—due to greater offspring philopatry—decreases evolved lifespans because selection against higher mortality is weaker if mortality reduces competition between relatives. It has been suggested that indirect fitness benefits that are gained post‐reproductively facilitate the evolution of extended postmenopausal lifespans, as found in humans and some species of whales, and thus higher relatedness between group members should lead to the evolution of longer lifespans (Lee 2008, 2003; Bourke 2007; Croft et al. 2015). However, in cooperative breeders, indirect fitness benefits can also be gained pre‐reproductively, because an individual's death reduces the waiting time for its relatives behind it in the queue, and this leads to a pre‐reproductive decline of the age‐specific force of selection. In line with this argumentation, relatively shorter lifespans evolve in our simulations when relatedness within breeding territories is high. Consistent with this result, a comparative study found that species‐specific survival in cooperatively breeding birds is positively correlated with species‐specific promiscuity, which in turn is negatively correlated with intragroup relatedness (Downing et al. 2015).

Our results demonstrate that timing of reproduction and kin structure within breeding territories are the most important drivers for the evolution of ageing in cooperative breeders. As helpers in cooperative breeders have the ability to breed later in their life, natural selection favors a delayed onset of senescence. In eusocial organisms, helping individuals belong to a worker caste and cannot inherit the breeder position later in their life (Crespi and Yanega 1995; Boomsma 2013; Boomsma and Gawne 2018). The absence of such direct fitness gains in late life can therefore partially explain why workers typically live much shorter than the reproductive castes of eusocial organisms (Kramer and Schaible 2013). Additionally, queens of eusocial insects typically only produce reproductive offspring after an extended period of producing workers and thus investing in colony growth (Kramer et al. 2016; Jaimes‐Nino et al. 2022). As our model predicts for cooperative breeders, such a delay of the production of reproductive offspring should impose strong selection against mortality even at advanced ages, potentially explaining the extraordinarily long lifespans of eusocial insect queens and kings (in termites) (Keller and Genoud 1997; Kramer et al. 2021; Kreider et al. 2021). Furthermore, following the logic from our model, we predict that, in eusocial organisms with high relatedness between queens and workers, workers should be more long‐lived than in species where relatedness between queens and workers is lower, for example, through multiple mating of queens (Kramer et al. 2021). This is because the strength of natural selection should scale positively with the amount of indirect fitness that can be gained through helping.

Overall, our model makes an important link between the evolutionary theory of ageing and kin selection theory, demonstrating that timing of reproduction and kin structure are the most important drivers for the evolution of ageing in cooperative breeders.

## CONFLICT OF INTEREST

The authors declare no conflict of interest.

## AUTHOR CONTRIBUTIONS

JJK and IP conceptualized and implemented the model. JJK and BHK analyzed the model. JJK, BHK, JK, and IP wrote the manuscript.

## DATA ARCHIVING

Simulation code and data analysis scripts are available under https://doi.org/10.34894/QPKQUO.

## Supporting information


**Figure S1**. The effect of mutation rate, mutation bias and mutational effect size on evolved lifespans.
**Figure S2**. The evolution of ageing in solitary and cooperative breeders.
**Figure S3**. Evolved lifespans over simulation time in the different solitary and cooperative breeding scenarios.
**Figure S4**. Evolved lifespans over simulation time in the different solitary and cooperative breeding scenarios.
**Figure S5**. Survivorship in the different solitary and cooperative breeding scenarios.
**Figure S6**. The effect of productivity on the evolution of ageing in cooperative breeders.
