# Supplementary material for: The evolution of ageing in cooperative breeders
Source: Evol Lett. 2022 Dec 1;6(6):450–9. doi: 10.1002/evl3.307 (PMC9783459; doi:10.1002/evl3.307)
Supplement: Supplementary file 1 — Figure S1. The effect of mutation rate, mutation bias and mutational effect size on evolved lifespans. Figure S2. The evolution of ageing in solitary and cooperative breeders. Figure S3. Evolved lifespans over simulation time in the different solitary and cooperative breeding scenarios. Figure S4. Evolved lifespans over simulation time in the different solitary and cooperative breeding scenarios. Figure S5. Survivorship in the different solitary and cooperative breeding scenarios. Figure S6. The effect of productivity on the evolution of ageing in cooperative breeders. [file EVL3-6-450-s001.pdf]

Supplementary materials

Title: The evolution of ageing in cooperative breeders

Authors: Jan J. Kreider, Boris H. Kramer, Jan Komdeur, Ido Pen

The ageing mechanism in the model

The evolutionary theory of ageing predicts that natural selection is more effective against mutations with negative fitness effects at young ages than at old ages. Consequently, mutations with negative fitness effects at older ages can accumulate by genetic drift (Medawar 1952; Williams 1957; Hamilton 1966). In our model, individuals possess two homologous genes for each age class. The average of the homologous genes determines an individual's survival probability to the next age class. We impose mutations with a mean effect size biased towards lower survival probabilities on these genes. Thus, mutations on average have a deleterious effect. This leads to the accumulation of mutations with deleterious survival effects if selection is too weak to remove these mutations. Fig. S1 shows how different mutation biases, mutational effect sizes and mutation rates affect the evolution of lifespans in the solitary model scenario. The interplay of these three parameters determines the "load" of mutations with negative survival effect imposed. In our model, we additionally assume that paternally and maternally-inherited genes are mutated simultaneously. This assumption also increases the mutational load since mutations with positive survival effects are on average accompanied by mutations with negative survival effects in other age classes.

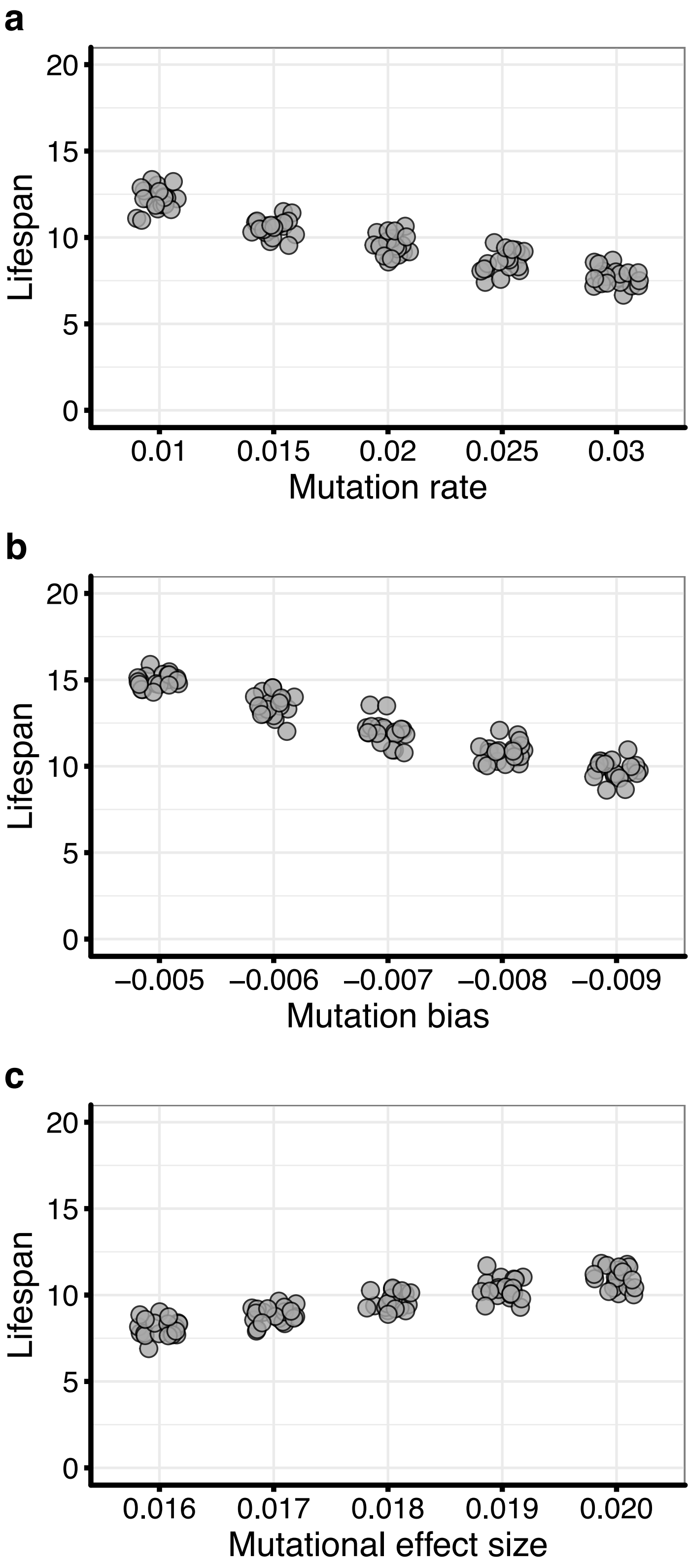

Figure S1. The effect of mutation rate, mutation bias and mutational effect size on evolved lifespans. (a) Larger mutation rates lead to the evolution of shorter lifespans because they increase the frequency of mutations with negative survival effects. (b) Stronger mutation bias leads to the evolution of shorter lifespans because it increases the negative effect of mutations on survival. (c) Larger mutational effect sizes lead to the evolution of longer lifespans because they allow for mutations with larger positive survival effects. Each data point is the mean evolved lifespan of a solitary population at the end of a replicate simulation ($n = 20$). Parameters: $a = 2.5$ (“maximum productivity”).

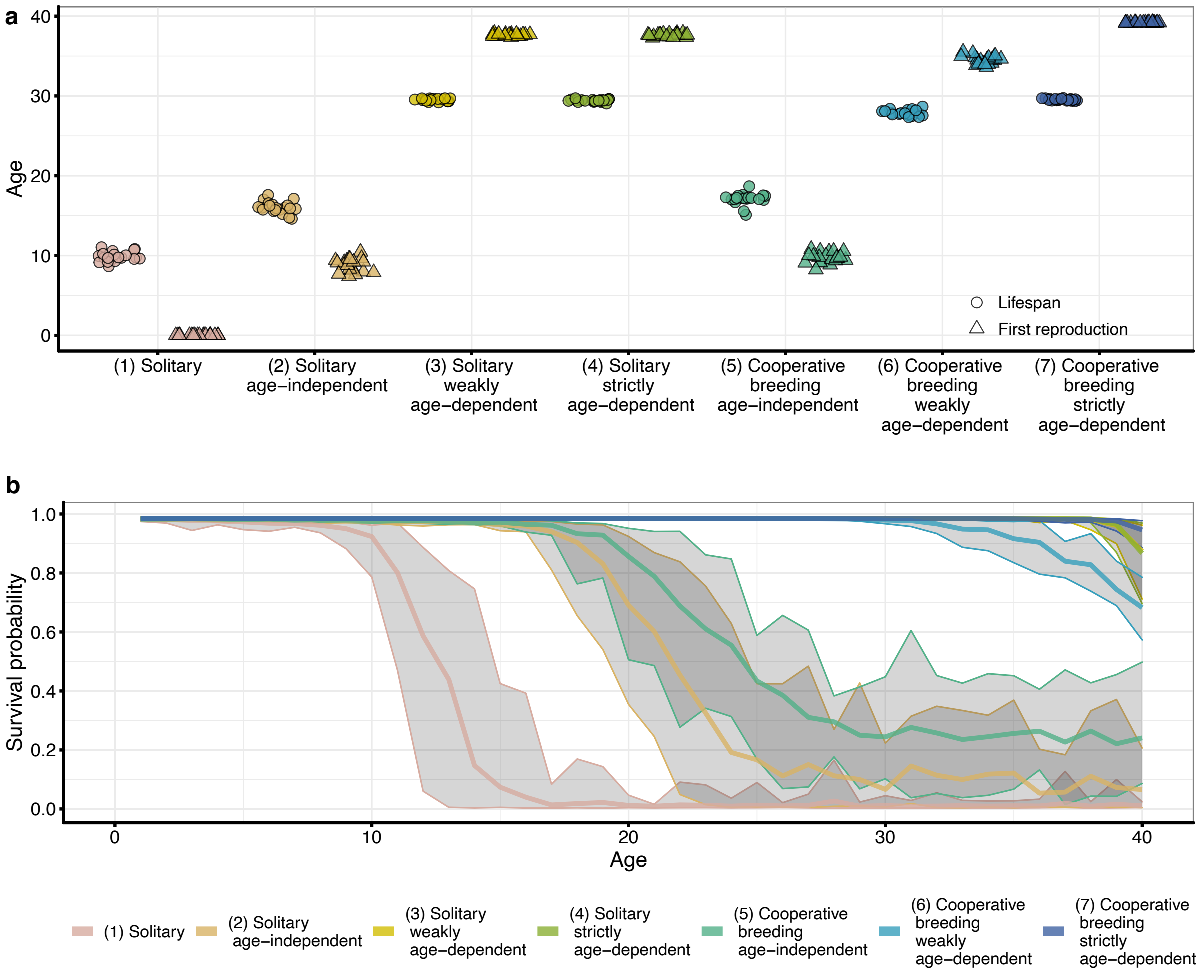

Figure S2. The evolution of ageing in solitary and cooperative breeders. (a) Evolved lifespans (circles) and age of first reproduction (triangles) for different solitary and cooperative breeding scenarios. Data points ($n = 20$) are the population mean at the end of a replicate simulation. (b) Survival probabilities for different solitary and cooperative breeding scenarios. Bold lines represent mean and grey shaded areas the range across replicate simulations. This figure is identical to Fig. 2 in the main manuscript only that here the maximum age is $c = 40$ instead of 20. Parameters: $d = 1.0$ (dispersal rate), $a = 2.5$ (“maximum productivity”).

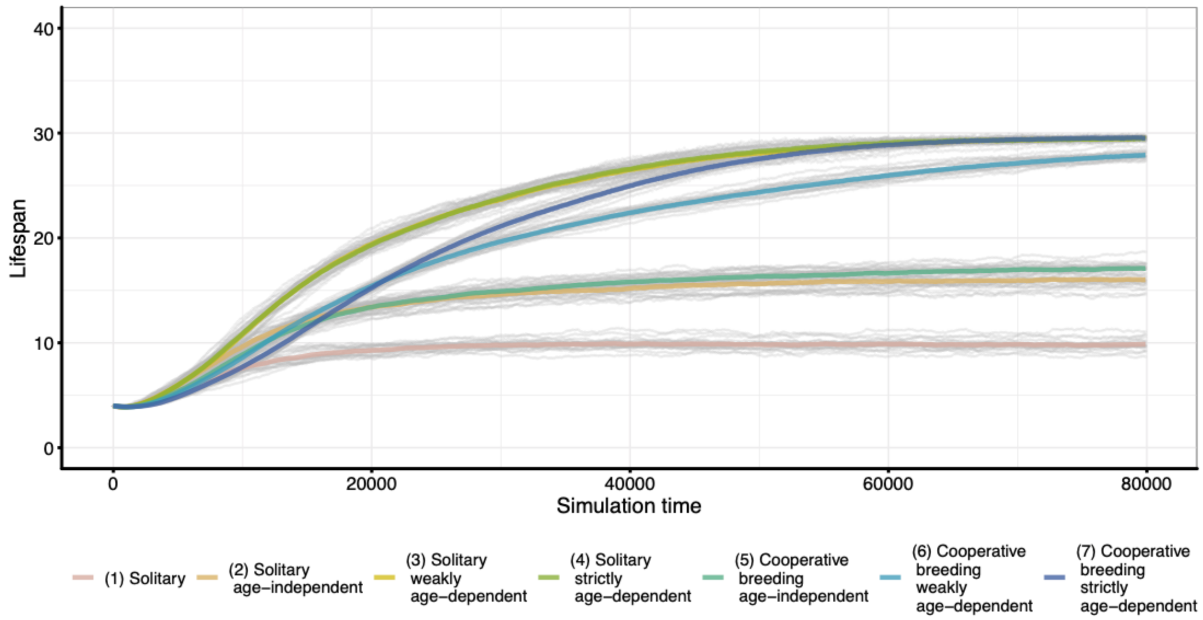

Figure S3. Evolved lifespans over simulation time in the different solitary and cooperative breeding scenarios. $c = 40$ (maximum age). Bold lines represent the mean lifespan across replicate simulations and grey lines individual replicate simulations. Each solitary and cooperative breeding scenario was run for 20 replicate simulations. Parameters: $d = 1.0$ (dispersal rate), $a = 2.5$ ("maximum productivity").

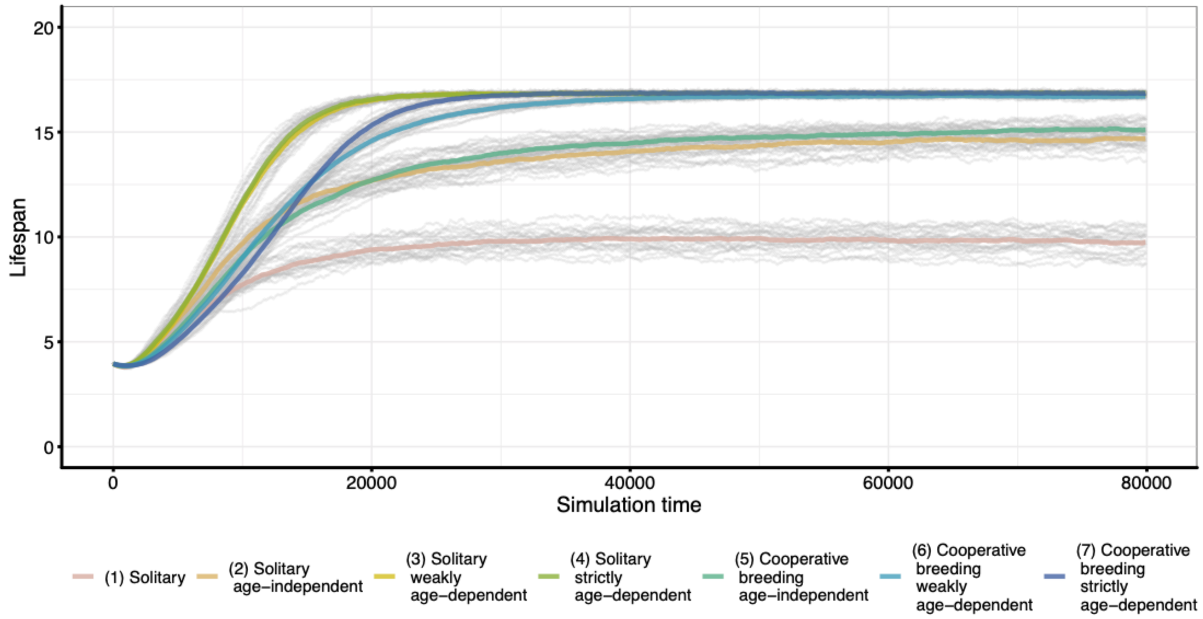

Figure S4. Evolved lifespans over simulation time in the different solitary and cooperative breeding scenarios. Bold lines represent the mean lifespan across replicate simulations and grey lines individual replicate simulations. Each solitary and cooperative breeding scenario was run

for 20 replicate simulations. Parameters: d = 1.0 (dispersal rate), a = 2.5 (“maximum productivity”).

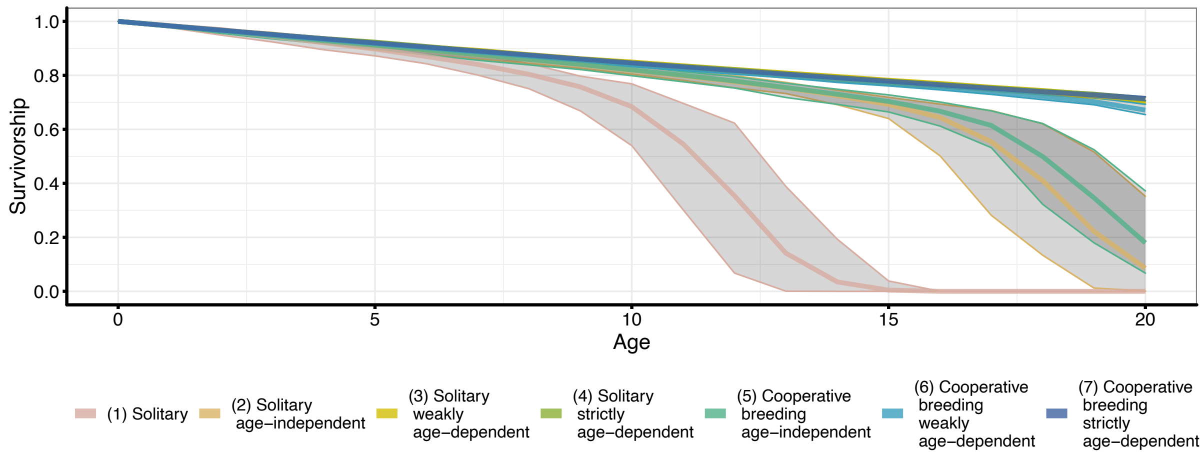

Figure S5. Survivorship in the different solitary and cooperative breeding scenarios. Bold lines represent the mean survivorship and grey areas the range across replicate simulations ($n = 20$). Survivorship was calculated as the cumulative product of the age-specific survival probabilities. Data from Fig. 2 of the main manuscript.

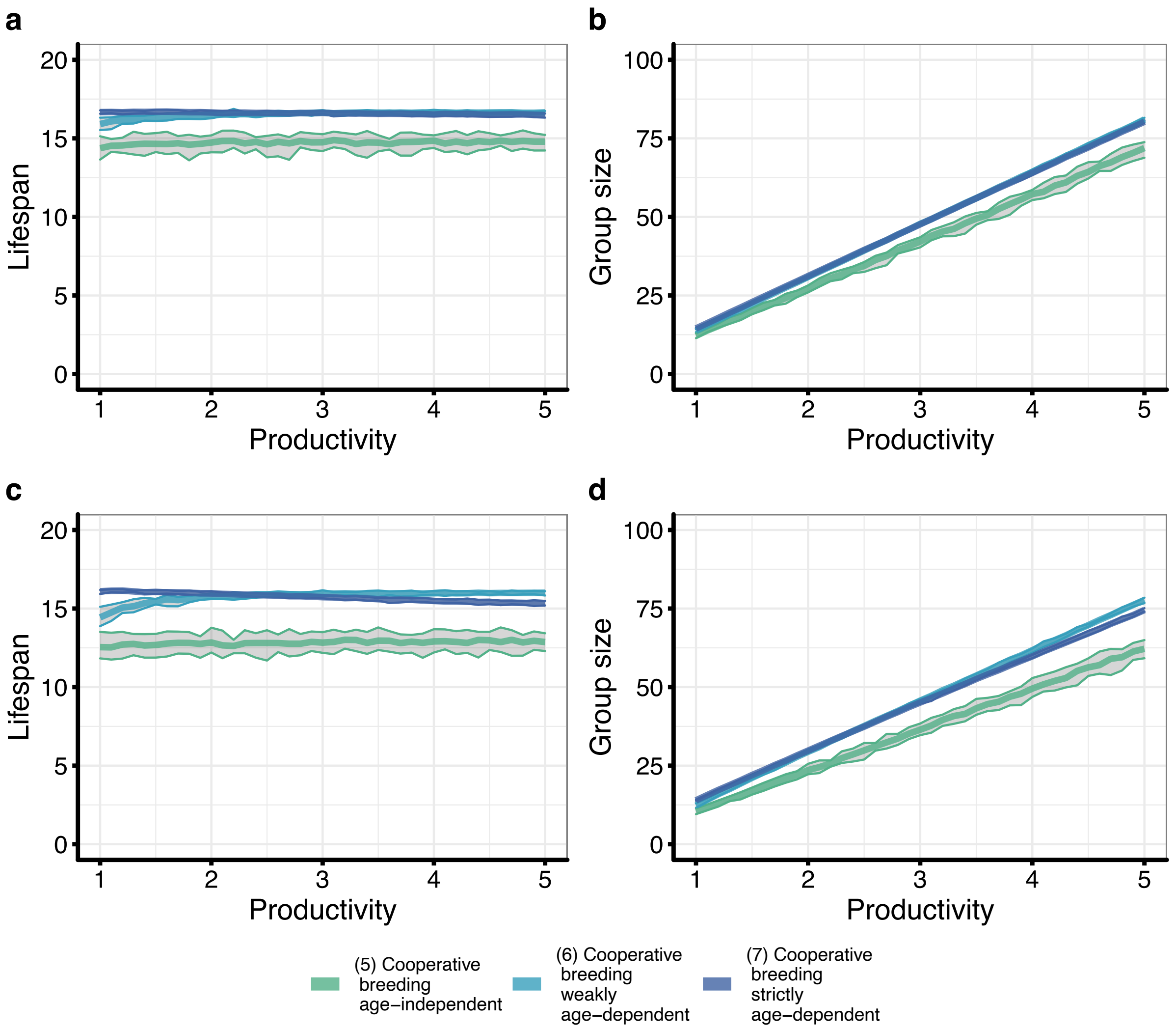

Figure S6. The effect of productivity on the evolution of ageing in cooperative breeders. (a + c) Evolved lifespans depending on "maximum productivity" in different cooperative breeding scenarios. "Maximum productivity" determines the number of offspring produced at large group sizes. (b + d) The effect of "maximum productivity" on group size. We ran each "maximum productivity" between $a = 1.0$ and $a = 5.0$ with steps of 0.1 ($n = 20$). (a + b) $d =$ 0.0, (c + d) $d = 1.0$ (dispersal rate). Bold lines represent the mean evolved lifespan and grey areas the range of evolved lifespans across replicate simulations.

Literature

Hamilton, W. D. 1966. The moulding of senescence by natural selection. Journal of Theoretical Biology 12:12–45.

Medawar, P. B. 1952. An unsolved problem of biology: an inaugural lecture delivered at university college, London, 6 December, 1951. H.K. Lewis and Company.

Williams, G. C. 1957. Pleiotropy, natural selection, and the evolution of senescence. Evolution 11:398–411.
